# Supplementary figures and images for: Development and characterization of a Gucy2d-cre mouse to selectively manipulate a subset of inhibitory spinal dorsal horn interneurons
Source: PLoS One. 2024 Mar 14;19(3):e0300282. doi: 10.1371/journal.pone.0300282 (PMC10939219; doi:10.1371/journal.pone.0300282)

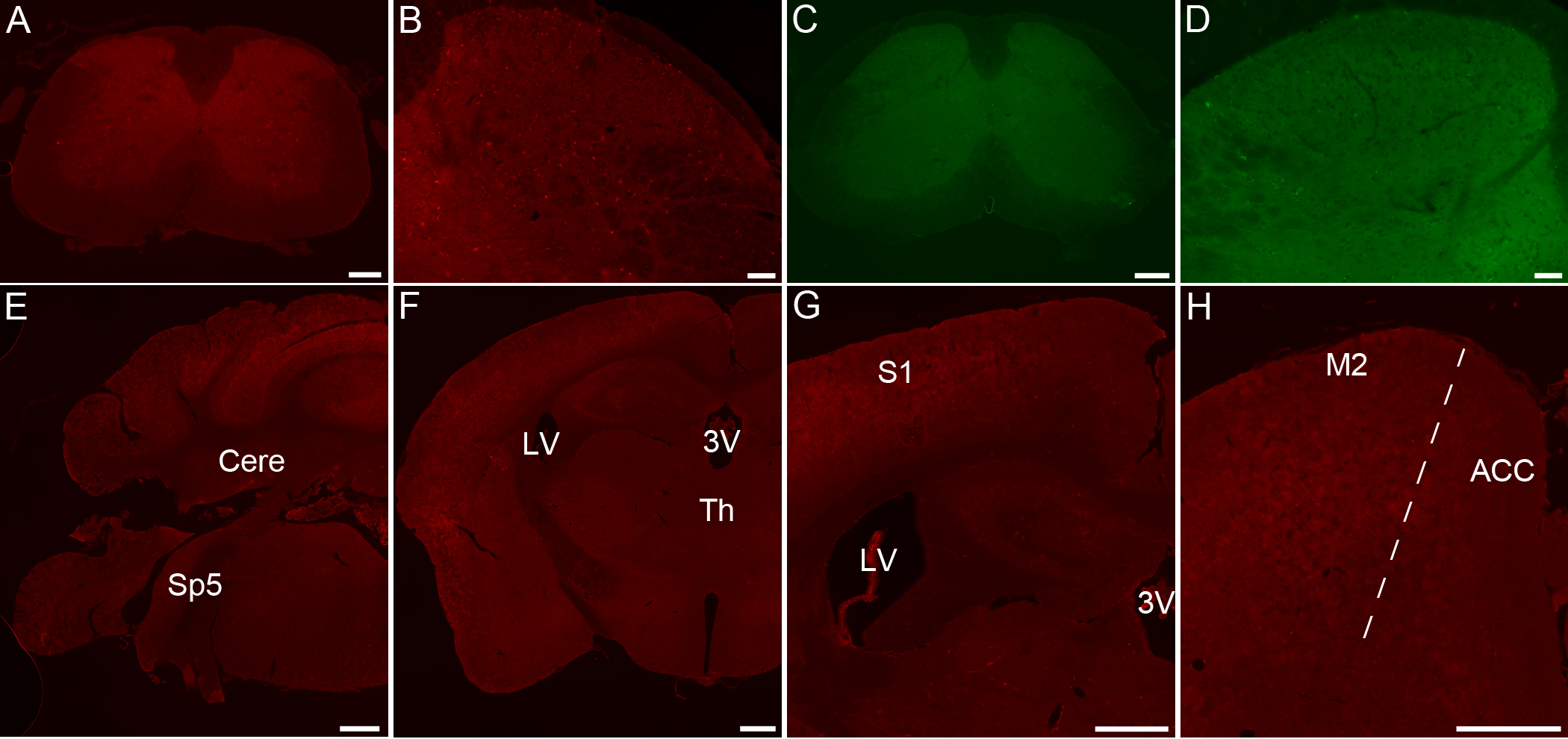

Supplement: S1 Fig — TdTomato expression is absent from the spinal cords of cre-negative Ai9 mice (A-B), and Sun1-GFP expression is absent from the spinal cords of cre-negative Sun1-GFP mice (C-D). Scale bars in A and C = 200 μm. Scale bars in B and D = 50 μm. TdTomato expression was likewise absent from the brains of cre-negative Ai9 mice, including the areas where Gucy2d-cre induced reporter expression in cre-positive mice: spinal trigeminal nucleus, cerebellum (Sp5, Cere; E), thalamus (Th; F), somatosensory cortex (S1; G), and anterior cingulate cortex (ACC; H). Scale bars in E-H = 500 μm. Other labeled landmarks include 3V: third ventricle, LV: lateral ventricle, M2: secondary motor cortex. (TIF) [file pone.0300282.s001.tif]

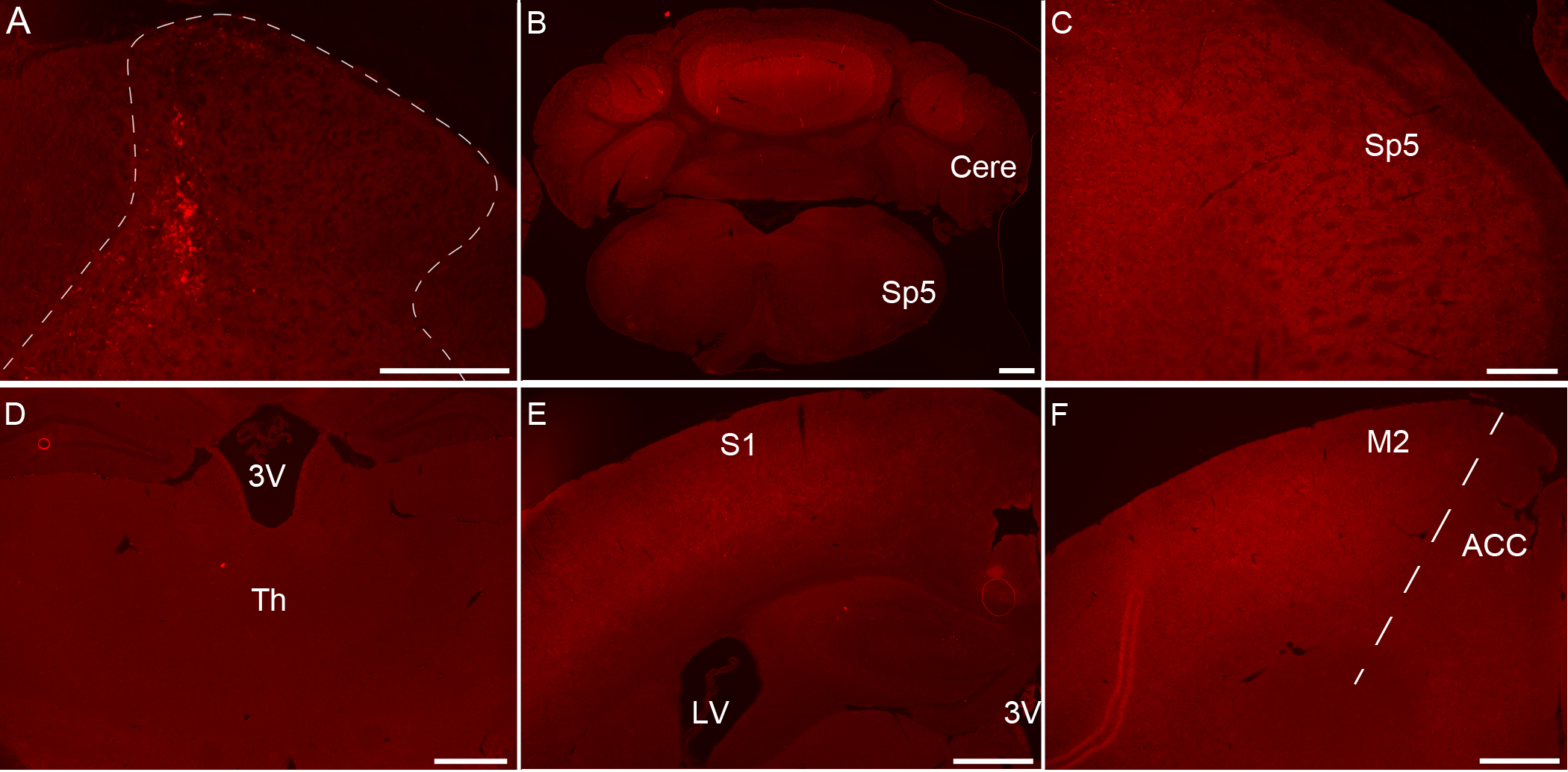

Supplement: S2 Fig — (A) Although autofluorescence is visible along the injection tract, intraspinal injection of AAV8-FLEX-CAG-tdTomato to cre-negative mice did not produce viral-driven reporter expression in dorsal horn neurons. Scale bar = 200 μm; dotted line = outline of dorsal horn. (B-F) Intravenous administration of AAV-PHP.eB-CAG-FLEX-tdTomato viral vector to cre-negative mice did not produce tdTomato labeling in the brain. Scale bars in B,D, E, F = 500 μm; Scale bar in C = 200 μm. Brain region and landmark abbreviations include cerebellum (Cere; B), spinal trigeminal nucleus (Sp5; B and C), thalamus (Th; D), somatosensory cortex (S1; E), anterior cingulate cortex (ACC; F), 3V: third ventricle, LV: lateral ventricle, M2: secondary motor cortex. (TIF) [file pone.0300282.s002.tif]
